# Do the primary surgical options for basic-type exotropia cause differences in distance-near discrepancy of recurrent exotropia after surgery?

**DOI:** 10.1371/journal.pone.0221268

**Published:** 2019-08-19

**Authors:** Kwan Hyuk Cho, Jinsoo Kim, Dong Gyu Choi, Joo Yeon Lee

**Affiliations:** 1 Department of Ophthalmology, Hallym University College of Medicine, Hallym University Sacred Heart Hospital, Anyang, South Korea; 2 Department of Ophthalmology, Hallym University College of Medicine, Hallym University Kangnam Sacred Heart Hospital, Seoul, South Korea; Faculty of Medicine, Cairo University, EGYPT

## Abstract

**Purpose:**

Most ophthalmologists appear to have no distinct preference between unilateral recess-resect (R&R) and bilateral lateral rectus (BLR) recessions to treat basic-type exotropia. This study aimed to determine whether differences in distance-near discrepancy and resultant exotropia types of recurrent exotropia following surgery for primary basic-type exotropia exist between the two surgical options.

**Methods:**

Ninety-three patients with recurrent exotropia following BLR recessions for basic-type exotropia (BLR group) and 95 following R&R for basic-type exotropia (R&R group) were included in this retrospective study. The exotropia types in recurrent exotropia were classified into three types according to distance-near discrepancy: basic, divergence-excess, and convergence-insufficiency. The BLR and R&R groups were compared.

**Results:**

After surgery for basic-type exotropia, the type composition changed differently in each group (p < 0.001). The basic-type of primary exotropia was more often maintained in recurrent exotropia in the R&R group than in the BLR group. The incidence of postoperative convergence-insufficiency type exotropia in the BLR group was 28.0% and 8.4% in the R&R group (p = 0.001). Postoperative near stereopsis and fusion control grade of distance deviation did not differ between the two groups (p > 0.05).

**Conclusions:**

Convergence-insufficiency type recurrent exotropia occurred more frequently after BLR recessions than after R&R for basic-type exotropia. The high rate of secondary convergence-insufficiency type exotropia after BLR recessions should be considered when clinicians select a surgical option to treat exotropia.

## Introduction

Surgical correction is the primary treatment for intermittent exotropia [1–3]. Selecting the appropriate surgical procedure for intermittent exotropia commonly depended on the exotropia type, which is classified according to distance-near discrepancy. Classically, the choice for basic-type exotropia with similar amount of deviation at distance and near was unilateral recess-resect (R&R), whereas for divergence-excess type exotropia with larger deviation at distance than at near, bilateral lateral rectus (BLR) recessions are preferred, and for convergence-insufficiency type exotropia with larger deviation at near than at distance, bilateral medial rectus resections [4–7]. The basis that warrants the choice of surgery for various exotropia types was that lateral rectus recession weakens distance divergence and medial rectus resection strengthens near convergence in the correction of exotropia [4–6, 8–10]. Anecdotally, most ophthalmologists currently appear to have no distinct preference between the two surgeries given the lack of clear principles. Both surgeries have demonstrated success rates in previous investigations, ranging from approximately 45% to 85% in basic-type exotropia [10–17]. Previous studies disagree as to the superior surgical procedure for each type of intermittent exotropia. Burian [4] and Kushner [9] obtained satisfactory surgical results using R&R for basic-type exotropia and BLR recessions for divergence-excess type exotropia. Some studies reported satisfactory results in both basic- and divergence-excess type exotropia treated using BLR recessions [8,10–11]. Choi et al [18] reported that long-term outcomes were better in eyes treated using BLR recessions for basic-type exotropia than in those undergoing R&R; however, Wang et al [13] reported the opposite.

Most studies that have compared the outcomes between BLR recessions and R&R were restricted to surgical success, and few investigations have examined the effect of surgical method on exotropia type with postoperative distance-near discrepancy [4,7,9–11,18]. The aim of the present study was to determine whether differences in distance-near discrepancy and resultant exotropia types of recurrent exotropia following surgery for primary basic-type exotropia exist between BLR recession and R&R. The main concern was the risk for development of convergence-insufficiency type recurrent exotropia after BLR recession if the BLR recession is expected to weaken the distance deviation rather than near deviation. Comparison of exotropia type in recurrent exotropia after surgery between the two surgical procedures is anticipated to provide a deeper understanding of the effect of each surgery on the distance and near deviation, and useful information to clinicians in choosing an appropriate surgical procedure for exotropia.

## Materials and methods

### Enrollment

The medical records of patients, who underwent BLR recession (BLR group) or R&R (R&R group) for basic-type intermittent exotropia between 2005 and 2012, were retrospectively reviewed. Patients who were diagnosed with recurrent exotropia with a distance angle > 10 prism diopters (PD) during the postoperative follow-up were identified. Patients who underwent vertical transposition of the horizontal rectus muscle or procedure for cyclovertical strabismus during the exotropia surgery, amblyopia, a history of ocular disease except strabismus, and a systemic anomaly, such as a neurological or congenital disorder, were excluded. The retrospective chart review was performed with approval of the Institutional Review Board of Hallym University Sacred Heart Hospital and adhered to the Declaration of Helsinki. Given the retrospective nature of the study and the use of anonymized patient data, requirements of informed consent were waived.

### Preoperative tests and surgery for exotropia

The exotropia operations were performed by two strabismologists at a single institution according to the surgical protocol modified from the surgical formula proposed by Wright [19] (Table 1). The surgeons had no insistent preference for BLR recession or R&R in basic-type exotropia with no amblyopia; the surgical option for the basic-type exotropia was routinely chosen by the surgeon. During routine care, we may encounter patients or their parents, who are anxious to operate on only one eye despite deviations in both eyes, particularly when the doctor suggests unilateral R&R, or in contrast, for those who wanted to avoid bilateral surgery. Unilateral or bilateral procedure for basic-type exotropia was determined according to the patients patients their parents, who are anxi. All surgical procedures were performed under general anesthesia, and adjustable suture techniques were not used. The angle of exodeviation was measured using the alternate prism cover test, with the accommodative targets for fixation at near (0.33 m) and at distance (6 m). The target angle for surgical dose and the basic-type of primary exotropia was determined using 1 h monocular occlusion before the patients were permitted to view with both eyes unoccluded.

**Table 1:** Surgical table of BLR recessions and R&R as primary surgery for basic-type intermittent exotropia.

Group	Distance deviation (PD)	LR recession (mm)	MR resection (mm)
**R&R group**	20	5.0	4.0
	25	6.0	5.0
	30	7.0	5.5
	35	7.5	6.0
	40	8.0	6.5
BLR group*	20	5.0	
	25	6.0	
	30	7.0	
	35	8.0	
	40	8.5	

BLR = bilateral lateral rectus recession; R&R = unilateral rectus recession-resection; PD = prism diopter

LR = lateral rectus muscel; MR = medial rectus muslce

*The recession was performed symmetrically on both lateral rectus muscles in the BLR group

The surgeons assessed and recorded the fusion control grade of the exodeviation at a fixation target of 6 m as follows: good control when fusion broke only after the cover test and resumed fusion rapidly without the need for refixation; fair control when a patient refixated to other targets to resume deviation control after the cover test; and poor control when a patient did not regain fusion despite refixation, and/or exhibited spontaneous deviation without the cover test or any event interfering with fusion [20,21]. For statistical analysis, “good” or “fair” from the medical record was defined as “A”, and “poor” as “B”. Near stereoacuity was measured using the Titmus-fly stereotest (Stereo Optical Co., Inc., IL, USA) at 0.33 m.

### Classification of exotropia types

The type of exotropia was determined based on a reference value according to the amount of distance-near discrepancy as follows [22]. In exotropia with a distance deviation ≥ 30 PD, the reference value was 10 PD; in exotropia with distance deviation < 30 PD, the reference value was one-third of the distance deviation. All primary exotropias were the basic-type, with the amount of distance-near discrepancy ≤ the reference value. The type of recurrent exotropia was determined as one of the three exotropia types as follows (Table 2): basic type, discrepancy ≤ reference value; divergence-excess-type, discrepancy > reference value, with the distance deviation exceeding near deviation; and convergence-insufficiency type, discrepancy > reference value, with near deviation exceeding the distance deviation. Divergence-excess type recurrent exotropia in the subjects was regarded as pseudo-divergence-excess type, which is a basic-type with tenacious proximal fusion, and we classified the recurrent exotropia type into two groups: basic/divergence-excess type recurrent exotropia; and convergence-insufficiency type exotropia.

**Table 2:** Classification system of exotropia types in recurrent exotropia.

Exotropia type	D ≥ 30	D < 30
**Basic**	D / N discrepancy ≤ 10	D / N discrepancy ≤ 1/3 of D
**Divergence-excess**	D–N > 10	D–N > 1/3 of D
**Convergence-insufficiency**	N–D > 10	N–D > 1/3 of D

D : Angle of exodeviation at distance (prism diopters)

N : Angle of exodeviation at near (prism diopters)

D / N discrepancy : The difference between angle of exodevation at distance and near

### Primary outcome measures

The primary outcome measure was the distribution of exotropia types in recurrent exotropia in the comparison between the BLR and R&R groups. Secondary outcome measures included differences in preoperative factors and postoperative outcomes according to the primary surgical options and exotropia type in recurrent exotropia.

### Statistical analysis

The χ^2^ test was used to compare exotropia type compositions of recurrent exotropia between the groups. The independent *t*-test was used to compare near stereopsis and fusion control between the groups. Additionally, Fisher’s exact test was used for comparison of preoperative exotropia type compositions between the groups. A probability value of < 0.05 was considered to be statistically significant. Statistical analysis was performed using SPSS version 21.0 (IBM Corporation, Armonk, NY, USA) for Windows (Microsoft Corporation, Redmond, WA, USA).

## Results

Ninety-three patients in the BLR group and 95 in the R&R group were enrolled in this study. There were no significant differences between the two groups in preoperative clinical findings, the mean length of time from the surgery to recurrence, or the duration of postoperative follow-up (Table 3).

**Table 3:** Patient characteristics in the BLR and R&R groups of basic-type intermittent XT.

	BLR group	R&R group	P value
Number of patients	93	95	
Sex (M:F)	45 : 48	49 : 46	0.88^a^
Age at surgery, mean, years	7.60 ± 4.23	6.02 ± 3.89	0.57^b^
Preoperative angle of deviation, mean±SD, prism diopters			
At distance	27.13 ± 9.51	30.21 ± 8.58	0.73^b^
At near	27.46 ± 11.81	30.54 ± 10.57	0.80^b^
At distance–At near	-0.37 ± 3.92	-0.77 ± 4.36	0.46^b^
Pseudodivergence-excess type : Basic type (N)	12 : 81	10 : 85	0.61^a^
Preoperative fusion control (A:B)	25 : 68	16:79	0.11^a^
Preoperative near stereopsis (Log of Seconds)	1.93 ± 0.41	2.05 ± 0.58	0.182^b^
Duration from time of surgery to recurrence, mean±SD, years	1.56 ± 1.84	1.98 ± 1.68	0.76^a^
Last postoperative follow-up, mean±SD, years	3.94 ± 2.61	4.02 ± 2.54	0.83^b^

XT = exotropia; BLR = bilateral lateral rectus recession; R&R = unilateral rectus recession-resection; A = good or fair fusion control; B = poor fusion control; SD = standard deviation

^a^P value by the chi-square test

^b^P value by the independent t-test

**Fig 1:**
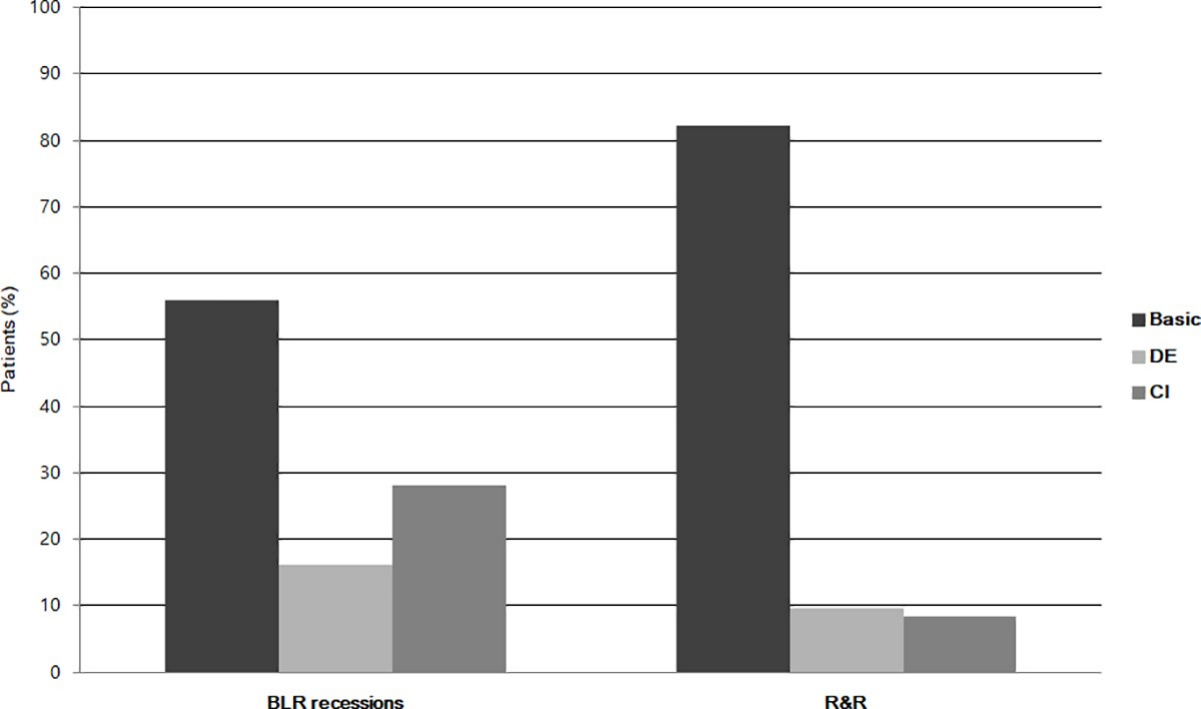
Bar graph showing the prevalence of the postoperative XT type between the BLR and R&R groups. The rate of postoperative CI-type XT in the BLR group (28.0%) was higher than that in the R&R group (8.4%) (p = 0.001). BLR = bilateral lateral rectus recession; R&R = unilateral rectus recession-resection; DE = divergence excess; CI = convergence insufficiency; XT = exotropia.

After surgery for treating basic-type exotropia, the exotropia type composition changed in each group. When compared the distributions of XT types between R&R group and BLF group, they were significantly different (p < 0.001) (Fig 1). In the BLR group, the exotropia type composition changed to 52 basic-type / 93 cases, 15 divergence-insufficiency type / 93, and 26 convergence-insufficiency type / 93, whereas it changed to 78 basic-type / 95 cases, 9 divergence-insufficiency type / 95, and 8 convergence-insufficiency type / 95 in the R&R group.

Postoperative near stereoacuity and fusion control of exodeviation improved after both types of surgery and did not differ between the two groups. The distance deviation angle in recurrent XT was greater in the R&R group than in the BLR group (p < 0.001) (Table 4).

**Table 4:** Postoperative motor-sensory outcomes in recurrent XT after BLR and R&R in basic-type intermittent XT.

	BLR group	R&R group	P value
Stereoacuity (Log of Seconds)	1.74 ± 0.18	1.80 ± 0.19	0.081 ^b^
Fusion control (A:B)	59 : 21	57 : 21	> 0.999^a^
Angle of distance deviation, mean±SD, prism diopters			
At distance	15.75 ± 4.01	20.04 ± 5.47	< 0.001^b^
At near	17.96 ± 7.89	21.41 ± 6.51	0.002^b^

XT = exotropia; BLR = bilateral lateral rectus recession; R&R = unilateral rectus recession-resection; A = good or fair fusion control; B = poor fusion control

^a^P value by the chi-square test

^b^P value by the independent t-test

When clinical features in patients with convergence-insufficiency type recurrent exotropia were compared with those with basic/divergence-excess type recurrent exotropia, no differences were found in preoperative factors or postoperative outcomes (p > 0.05 for all) (Table 5).

**Table 5:** Comparison of the clinical variables of convergence-insufficiency-type recurrent exotropia and basic/divergence-excess-type recurrent exotropia.

Clinical features	CI-type recurrent XT	Basic/DE-type recurrent XT	P value
Preoperative			
Fusion control (A:B) (N)	9 : 25	32 : 122	0.494 ^a^
Distance deviation angle, mean±SD, prism diopters	27.73 ± 8.34	29.18 ± 9.72	0.461 ^b^
Stereoacuity, mean±SD, Log of Seconds	1.93 ± 0.42	2.00 ± 0.51	0.490 ^b^
Age at surgery, mean±SD, years	7.11 ± 3.89	7.63 ± 6.99	0.401 ^b^
Pseudodivergence excess : Basic type	3 : 31	19 : 135	0.770^c^
Postoperative			
Fusion control (A:B) (N)	24 : 7	92 : 35	0.656 ^a^
Distance deviation angle, mean±SD, prism diopters	15.28 ± 3.34	18.66 ± 5.44	0.001 ^b^
Stereoacuity, mean±SD, Log of Seconds	1.74 ± 0.18	1.79 ± 0.18	0.134 ^b^
Duration from time of surgery to recurrence, mean±SD, years	1.65 ± 1.81	1.77 ± 2.25	0.861 ^b^
Last postoperative follow-up, mean±SD, years	3.86 ± 1.91	4.12 ± 3.01	0.517 ^b^

CI = convergence insufficiency; XT = exotropia; DE = divergence excess; A = good or fair fusion control; B = poor fusion control

^a^P value by the chi-square test

^b^P value by the independent t-test

^c^P value by the Fisher’s exact test

## Discussion

The change in the exotropia type demonstrated a different pattern after BLR recession versus R&R. The basic-type of primary exotropia was maintained more often after R&R than after BLR recession. The incidence of convergence-insufficiency type recurrent exotropia after BLR recession for basic-type primary exotropia was 28.0% (26/93). This was significantly higher than the incidence of convergence-insufficiency type recurrent exotropia after R&R, which was 8.4% (8/95). This result suggested that distance divergence is more affected than near deviation in BLR recession, and the surgical method used may influence future distance-near discrepancy. Moreover, the distance deviation of recurrent exotropia in the BLR group was smaller than that in the R&R group, even though there were no differences in length of time to recurrence or total follow-up period between the two groups. This result may suggest the superior suppression effect of BLR recession on the distance exodeviation relative to R&R. We believe that this effect is related to the lower long-term recurrence of exotropia after BLR recession than after R&R reported in some previous studies [12,18]. Choi et al [18] reported that the surgical results were not different between BLR recession and R&R at 2 years after surgery; however, the final long-term outcomes were better in BLR recessions than in R&R. The researchers explained that the long-term difference in outcomes was related to the low recurrence of distance exodeviation during the postoperative period after BLR recession. Furthermore, in a study by Xain et al [12], the motor outcomes after R&R were better than those after BLR recession at 6 months after surgery, while the 3-year outcomes were better after BLR recession due to lower recurrence rates. These previous studies, however, did not assess the exotropia type or the risk for convergence-insufficiency type exotropia. In addition, in the present study, near stereoacuity and fusion control of distance exodeviation in the recurrent exotropia did not significantly differ between the two groups, despite these differences existing in distance and near deviation.

In our results, some cases also changed into divergence-excess type recurrent exotropia from the primary basic-type exotropia; however, not in the true sense of type conversion. Pseudo-divergence-excess type can be differentiated from true-divergence-excess type through the occlusion test in cases of significant distance-near discrepancy with distance deviation exceeding near deviation. When the near deviation increases and becomes the basic-type without distance-near discrepancy after monocular occlusion to eliminate tenacious proximal fusion, the case can be confirmed as pseudo-divergence-excess type [4,5]. All patients enrolled in the present study had basic-type exotropia before surgery. We performed the occlusion test preoperatively in the primary exotropia, but it was not routinely performed in cases of recurrent exotropia. However, we believe that the recurrent exotropia with divergence-excess type in this study should be pseudo-divergence-excess type because true-divergence-excess type has an inherent large proximal convergence [4,5], and a rare possibility exists that the true-divergence-excess type recurrent exotropia in this study was a new occurrence postoperatively from preoperative basic-type exotropia. Furthermore, we speculate the reason why this postoperative divergence-excess-type (regarded as pseudo-divergence-excess-type) developed more in the BLR group (15/93) than in R&R group (9/95) was the smaller angle of deviation in the BLR group than in the R&R group, thus facilitating tenacious fusion.

In this study, the three types of exotropia were determined according to distance-near discrepancy. The reference value for distance-near discrepancy varies from 5 PD to 15 PD across studies. As such, the significant reference value for the type of exotropia remains controversial [4,6,11,17,23]. This study included patients with recurrent exotropia after surgery with a smaller angle than the primary exotropia; and a reference value of 10 PD, which is generally used, would be a relatively large difference in small angle exotropia. To avoid underestimating differences in distance-near discrepancy between the two surgery groups, we refined the reference value according to the distance deviation. The reference value was defined as one-third of the distance angle in cases with a distance angle < 30 PD, and 10 PD in cases with a distance angle ≥ 30 PD [22].

Convergence-insufficiency-type exotropia is often considered to be unfavorable, and symptoms, such as headache, diplopia, blurred vision and asthenopia, can be associated with it [4–5, 24–27]. In this study, however, near stereoacuity did not significantly differ between the BLR and R&R groups, and convergence-insufficiency type recurrent exotropia exhibited good stereoacuity, similar to basic/divergence-excess type recurrent exotropia. This meant that BLR recession for intermittent exotropia could cause convergence-insufficiency type recurrent exotropia but did not weaken the near fusion mechanism. We suggest, however, that BLR recession be cautiously used in patients at risk for weak fusional mechanism at near deviation, although the postoperative convergence-insufficiency type did not demonstrate deteriorated clinical outcome in this study.

To our knowledge, this was the first investigation to compare secondary exotropia types in recurrent exotropia after BLR recession versus R&R for basic-type exotropia. Limitations of this study include the relatively small sample size and its single-institution design, and the fact that the determination of fusion control of distance exodeviation was based on the surgeon’s inspection in clinical practice. Because this study was retrospective in nature and involved a chart review, the latter was not a controlled factor. An additional limitation of this retrospective study was the lack of a postoperative patch test to confirm our estimation that the postoperative divergence-exotropia type should be the pseudo-divergence-excess type, as discussed above.

In summary, the change in exotropia type according to distance-near discrepancy demonstrated a different pattern after BLR recession versus R&R. The basic-type in primary exotropia was maintained more in recurrent exotropia after R&R than in that after BLR recession. Postoperative convergence-insufficiency type exotropia occurred more frequently after BLR recession than after R&R for basic-type exotropia. On the other hand, the amount of distance deviation in recurrent exotropia was smaller after BLR recession than after R&R, and near stereopsis was not deteriorated in the convergence-insufficiency type recurrent exotropia. The risk for the occurrence of a high rate of convergence-insufficiency type exotropia after BLR recession, however, should be considered when clinicians select a surgical option to treat exotropia.
